# A Zinc Polyphenolic Compound Increases Maize Resistance Against Infection by *Bipolaris maydis*

**DOI:** 10.3390/plants14010077

**Published:** 2024-12-30

**Authors:** Luis Felipe Lata-Tenesaca, Marcos José Barbosa Oliveira, Aline Vieira Barros, Leandro Castro Silva, João Américo Wordell Filho, Fabrício Ávila Rodrigues

**Affiliations:** 1 Laboratório da Interação Planta-Patógeno, Departamento de Fitopatologia, Universidade Federal de Viçosa, Viçosa 36570-900, Minas Gerais, Brazil; luis.felipe1@ufv.br (L.F.L.-T.); marcos.j.oliveira@ufv.br (M.J.B.O.); alinevieiradebarros@gmail.com (A.V.B.); leandrocsilva1989@gmail.com (L.C.S.); 2 Empresa de Pesquisa Agropecuária e Extensão Rural de Santa Catarina (Epagri/Cepaf), Avenida Servidão Ferdinando Tusset, s/n, Bairro São Cristovão, Caixa Postal 791, Chapecó 89801-970, Santa Catarina, Brazil; wordell@epagri.sc.gov.br

**Keywords:** antioxidative metabolism, cell defense reactions, induced resistance, mineral plant nutrition, photosynthesis

## Abstract

Maize leaf blight (MLB), caused by the fungus *Bipolaris maydis*, is an important disease affecting maize production. In order to minimize the use of fungicides in agriculture, nutrient-based resistance inducers may become a promising alternative to manage MLB. The goal of this study was to investigate the potential of Semia^®^ (zinc (20%) complexed with a plant-derived pool of polyphenols (10%)) to hamper the infection of maize leaves by *B. maydis* by analyzing their photosynthetic performance and carbohydrate and antioxidative metabolism, as well as the expression of defense-related genes. Plants were sprayed with water (control) or Semia^®^ (referred to as induced resistance (IR) stimulus hereafter) and not inoculated or inoculated with *B. maydis*. The mycelial growth and conidium germination were significantly reduced by the IR stimulus in vitro. The MLB severity was significantly reduced by 76% for IR-stimulus-sprayed plants compared to plants from the control treatment. For infected and IR-stimulus-sprayed plants, the glucose, fructose, sucrose, and starch concentrations were significantly higher compared to inoculated plants from the control treatment. The activity levels of superoxide dismutase, ascorbate peroxidase, catalase, and glutathione reductase were significantly higher for the IR-stimulus-sprayed plants compared to plants from the control treatment. Less impairment on the photosynthetic apparatus (higher values for leaf gas exchange (rates of net CO_2_ assimilation, stomatal conductance to water vapor, and transpiration) and chlorophyll *a* fluorescence (variable-to-maximum Chl *a* fluorescence ratio, photochemical yield, and yield for dissipation by down-regulation) parameters)) along with a preserved pool of chlorophyll *a*+*b* and carotenoids were noticed for infected and IR-stimulus-sprayed plants compared to infected plants from the control treatment. The defense-related genes *IGL*, *CHS02*, *PR1*, *PAL3*, *CHI*, and *GLU* were strongly up-regulated in the leaves of IR-stimulus-sprayed and infected plants compared to infected plants from the control treatment. These findings highlight the potential of using this IR stimulus for MLB management.

## 1. Introduction

Maize (*Zea mays* L.) is one of the main staple crops, both agriculturally and economically, guaranteeing global food and nutritional security [1]. Although maize is more adaptable to abiotic stresses than other cereals, infection by pathogens has been the main obstacle limiting plant growth and to achieving greater yields [2]. The occurrence of maize leaf blight (MLB) epidemics, caused by the fungus *Bipolaris maydis* (Y. Nisik. and Miyake) Shoemaker, has contributed significantly to yield losses due to photosynthetic impairment, reduced plant growth, and less allocation of assimilates from leaves to the developing grains [3,4].

Approaches involving the use of fungicide spray, hybrids with higher levels of basal resistance, plant genome editing using the CRISPR/Cas9 system, protective formulations using nanotechnology, biological control methods (e.g., *Bacillus cereus* and *Trichoderma atroviride*), crop rotation, and balanced plant nutrition (e.g., nitrogen, potassium, and silicon) are some of the available strategies for MLB management [3,4]. However, other control strategies that will help the growers, especially those from developing countries, to reduce the cost of fungicides and their harmful effects on human health and the environment need to be investigated towards more sustainable agriculture. Thinking about more sustainable maize production, resistance inducers may become an environmentally friendly alternative for MLB management, especially if combined with fungicides and other biological control options to reduce the chemical inputs without losing efficacy. It is well known that plants exposed to resistance inducers of an abiotic or biotic nature efficiently activate defense reactions that will hinder the infection by pathogens of different lifestyles [5,6,7]. During the induced resistance, which can be assigned as systemic acquired resistance (SAR) or induced systemic resistance (ISR), a plethora of signaling pathways mediated by hormones (e.g., salicylic acid (SA), jasmonic acid (JA), and ethylene (ET)), with the co-participation of mobile signals (e.g., glycerol-3-phosphate, azelaic acid, pipecolic acid, and N-hydroxy-pipecolic acid), will take place to coordinate the temporal and spatial action of different defense mechanisms [7,8]. It is still a debate in the scientific community whether the inducers of resistance should or should not display a fungistatic effect against the development of pathogens (e.g., spore germination and mycelial growth). For instance, many inducers of resistance of an abiotic nature such as the well-known acibenzolar-S-methyl are capable of affecting the viability of pathogen structures in in vitro assays [6,7]. Interestingly, the spraying of nutrient-based resistance inducers has been efficient in reducing the intensity levels of diverse fungal diseases in crops such as soybean, tomato, and potato [9,10,11]. Zinc (Zn) is involved in the signaling of SAR and ISR, which allow plants to activate different cascades of cell defense reactions at the infection sites of pathogens, as well as improving the integrity and permeability of the membrane cell wall [12]. Maize plants sprayed with Zn–chitosan nanoparticles showed reduced symptoms caused by infections of *Curvularia lunata* and *Bipolaris sorokiniana* as a result of boosted antioxidant and defense systems and increased production of lignin in the infected leaf tissues [13]. Additionally, the grain yield increased and the grains had a higher content of Zn for the plants sprayed with Zn–chitosan nanoparticles [13].

The hypothesis that spraying maize leaves with a zinc polyphenolic compound could increase their level of basal resistance (e.g., preservation of the photosynthetic apparatus, boosted defense reactions, and a better response of the antioxidant enzymes) against infection by *B. maydis* was investigated in this study using different physiological, biochemical, and molecular approaches. In this regard, plants not sprayed or sprayed with this compound and not infected or infected by *B. maydis* were analyzed for their photosynthetic performance and carbohydrate and antioxidative metabolism, as well as their expression of defense-related genes.

## 2. Materials and Methods

### 2.1. In Vitro Assays

Different volumes of a stock solution of Semia^®^ (40 mL of this product per liter of sterile deionized water) (nitrogen (1%) and zinc (20%) complexed with a plant-derived pool of polyphenols (10%); FertiGlobal, Larderello, Italy) were mixed with 1 mL of a conidial suspension of *B. maydis* (5 × 10^4^ conidia/mL) to obtain suspensions containing 1, 2.5, 5, 10, and 15 mL of Semia^®^/L. A total of 100 µL of conidial suspension containing the different concentrations of Semia^®^ was transferred to a glass slide that was covered with a coverslip and also to a Petri dish containing 20 mL of potato dextrose agar (PDA) medium. The conidial suspension of *B. maydis* without Semia^®^ corresponded to the control treatment. The conidial suspension was homogeneously distributed over the PDA medium using a Drigalski glass spatula. The glass slides and the Petri dishes were transferred to a growth chamber (25 °C and a 12 h light–12 h dark photoperiod). Each glass slide and Petri dish received 5 μL of lactofuchsin after 12 h to stop the conidium germination. One hundred conidia were randomly examined in each glass slide or Petri dish under a light microscope (Carl Zeiss AxioImager A1, Oberkochen, Germany) using a bright field at 400× magnification. Details of the conidium germination were acquired digitally (model AxioCam HR and Axion Vision software v. 4.8.1; Zeiss AG, Oberkochen, Germany). A conidium with a germ tube larger than its diameter was considered germinated. The percentage of conidia germination was calculated per replication of each treatment.

### 2.2. Plant Growth

Maize seeds from the cultivar Colorado SCS 156 (EPAGRI, Crituba, Santa Catarina, Brazil), susceptible to *B. maydis*, were sown in plastic pots containing 2 kg of Tropstrato^®^ (mixture of pine bark, peat, and expanded vermiculite (1:1:1); Vida Verde Company, São Paulo, Brazil) substrate. A total of 1.63 g of calcium phosphate was added to each pot to provide phosphorus to the plants before sowing. After the seedlings’ emergence (≈five days), two plants were kept per pot. The plants were fertilized with nutrient solution (100 mL per pot twice a week) prepared according to Hoagland and Arnon [14], with a few modifications, as follows: 2.6 mM KCl, 0.6 mM K_2_SO_4_, 1.2 mM MgSO_4_, 1.0 mM CH_4_N_2_O, 1.2 mM NH_4_NO_3_, 0.0002 mM (NH_4_)_6_Mo_7_O_24_, 0.03 mM H_3_BO_4_, 0.04 mM ZnSO_4_, 0.01 mM CuSO_4_, 0.03 mM MnCl_2_, 0.015 mM FeSO_4_, and 0.015 mM ethylenediaminetetraacetic acid disodium (EDTA). The plants were kept in the greenhouse (temperature of 28 ± 5 °C, relative humidity of 80 ± 5%, and natural photosynthetically active radiation (PAR) of 900 ± 15 μmol photons m^−2^ s^−1^ measured at midday).

### 2.3. Foliar Spray of Plants with Semia^®^

Maize plants (V6 growth stage, 30 days after seedlings emergence) were sprayed with a solution of Semia^®^ (5 mL per liter of sterile deionized water; 12.5 mL of solution per plant in each pot) 48 h before inoculation with *B. maydis* with the aid of a VL Airbrush atomizer (Paasche Airbrush Co., Chicago, IL, USA). This treatment will be referred to as an induced resistance (IR) stimulus hereafter, according to the criteria proposed by Kesel et al. [8]. Plants sprayed with water served as the control treatment.

### 2.4. Plant Inoculation with B. maydis

Pieces of filter paper (≈1 mm^2^) containing fungal mycelia of the monosporic isolate of *B. maydis* UFV-DPF-*Bm*12 were transferred to Petri dishes containing PDA medium. The dishes were placed inside of a growth chamber (25 °C and a photoperiod of 12 h of light and 12 h of dark) for fungal growth and conidium production for 15 days. The conidia were collected from each dish using sterile deionized water (0.01% Tween 20 and 0.5% gelatin (*w*/*v*)) and the conidial suspension was calibrated to 1 × 10^3^ conidia/mL using a Neubauer chamber. The plants were inoculated with the conidial suspension of *B. maydis* using a VL Airbrush atomizer and maintained inside a mist growth chamber (25 °C and relative humidity of 90 ± 5%) for 24 h. After this period, the plants were transferred to a greenhouse (28 ± 2 °C, relative humidity of 80 ± 5%, and natural PAR of 900 ± 15 μmol photons m^−2^ s^−1^ measured at midday) until the end of the experiments.

### 2.5. Evaluation of MLB Severity

The fifth expanded leaf, from base to top, of each plant per replication of each treatment was collected 156 h after inoculation (hai) and scanned at a 600 dpi resolution. The images from all leaves were processed using the QUANT software version 1.0 [15] to obtain the severity values. At 156 hai, lesions were very well developed in the leaves of plants from the control treatment.

### 2.6. Determining the Foliar Concentrations of Zn and N

At the end of the experiment (156 hai), the fifth and sixth leaves, from base to top, of each plant per replication of each treatment were collected, washed in deionized water, dried for 72 h at 65 °C, and ground in a ball mill (TECNAL TE 350, São Paulo, Brazil) for 2 min. The leaf samples were digested with a perchloric acid and nitric acid (1:2) solution following the readings in an atomic absorption spectrophotometer to determine the Zn concentration [16]. For the N concentration, the leaf samples were digested with sulfuric acid and subsequently oxidized with hydrogen peroxide. Aliquots from the extract were reacted with potassium chloride and Nessler reagent with a reading at 440 nm in a spectrophotometer.

### 2.7. Determining the Leaf Gas Exchange Parameters

The net carbon assimilation rate (*A*), stomatal conductance to water vapor (*g*_s_), internal CO_2_ concentration (*C*_i_), and transpiration rate (*E*) were measured on the fifth leaf, from base to top, of each plant per replication of each treatment at 12, 60, 108, and 156 hai from 09:00 h to 12:00 using a portable open-system infrared gas analyzer (LI-6400, LI-COR Inc., Lincoln, NE, USA). These time-points corresponded to the infection process of *B. maydis* from penetration (12 hai) to leaf tissue colonization (60 hai) and symptom development (from 108 to 156 hai) [17]. These parameters were evaluated on the fifth leaves of non-inoculated plants at the same evaluation times mentioned above. All measurements were carried out under the following conditions: leaf temperature of 25°C, chamber CO_2_ concentration of 420 ppm, PAR of 1200 μmol m^−2^ s^−1^, and amount of blue light set to 10% of PAR to optimize the stomatal aperture [4].

### 2.8. Evaluation of Chl a Fluorescence Parameters

The Imaging-PAM fluorometer and the Imaging Win software MAXI version (Heinz Walz GmbH, Eichenring, Germany) were used to obtain the images of Chl *a* fluorescence parameters [variable-to-maximum chlorophyll *a* fluorescence ratio (*F*_v_/*F*_m_), photochemical yield (Y(II)), yield for dissipation by down-regulation (Y(NPQ)), and yield for non-regulated dissipation (Y(NO))] on the fifth leaf, from base to top, of each plant not inoculated or inoculated with *B. maydis* at 12, 60, 108, and 156 hai per replication of each treatment according to the methodology described by Fagundes-Nacarath et al. [15]. These time-points were selected as mentioned above.

### 2.9. Determining the Photosynthetic Pigment Concentration

The concentrations of Chl *a*, Chl *b*, and carotenoids were quantified on the leaves used to obtain the images of Chl *a* fluorescence parameters. The leaf tissue (0.1 g) was ground in liquid nitrogen using a vibration ball mill (Retsch, Haan, Germany) and the fine powder was homogenized with 700 µL of methanol. The supernatant was used to quantify the concentrations of Chl *a*, Chl *b*, and carotenoids with readings performed in a spectrophotometer at 470, 653, and 666 nm, respectively, using a saturated solution of methanol as a blank [18].

### 2.10. Histochemical Detection of Lipid Peroxidation, Membrane Damage, Hydrogen Peroxide (H_2_O_2_), and Superoxide Anion Radical (O_2_^•−^) in Leaf Tissues

The fifth leaf, from base to top, of each plant per replication of each treatment was collected from both non-inoculated and inoculated plants at 156 hai. The lipid peroxidation, membrane damage, H_2_O_2,_ and O_2_^•−^ were visualized using Schiff, Evans’ blue, 3,3′-diaminobenzidine tetrahydrochloride, and nitro blue tetrazolium solutions, respectively, following the procedures described by Silva et al. [10].

### 2.11. Biochemical Assays and Gene Expression Analysis

The fifth leaf, from base to top, of each plant per replication of each treatment was collected at 12, 60, 108, and 156 hai. Leaves from non-inoculated plants were sampled at these same evaluation times. These time-points were selected as mentioned above. The leaf samples were kept in liquid nitrogen during sampling and stored in an ultra-freezer (−80 °C) until further analysis.

#### 2.11.1. Determining Sugar and Starch Concentrations

The leaf tissue (0.1 g) was ground into a fine powder as described above and mixed with 700 µL of methanol at 80 °C for 20 min. The sugars and starch were extracted according to Medeiros et al. [19]. The glucose, fructose, and sucrose contents were determined in the soluble phase of the methanolic solution and the pellet was used to quantify the starch following the procedures described by Fernie [20].

#### 2.11.2. Determining the Malondialdehyde (MDA) Concentration

The leaf tissue (0.1 g) was ground as described above and homogenized in 2 mL of trichloroacetic acid solution (0.1% (*w*/*v*)) following centrifugation at 12,000× *g* at 4 °C for 15 min. A total of 750 µL of thiobarbituric acid solution was added to 250 µL of the supernatant followed by homogenization in a thermomixer at 95 °C for 30 min. The samples were centrifuged at 9000× *g* for 10 min and the absorbance readings were taken at 600 and 532 nm [18].

#### 2.11.3. Determining H_2_O_2_ and O_2_^•−^ Concentrations

The leaf tissue (0.1 g) was ground as described above and homogenized in 1 mL of solution containing potassium phosphate buffer (50 mM, pH 6.5) and hydroxylamine (1 mM). The homogenate was centrifuged at 10,000× *g* at 4 °C for 15 min. The supernatant was used to determine the H_2_O_2_ concentration according to Dias et al. [21]. The leaf tissue (0.1 g) was ground as described above and the fine powder was homogenized in 1 mL of solution containing potassium phosphate buffer (100 mM, pH 7.2) and sodium diethyldithiocarbamate (1 mM). The homogenate was centrifuged at 22,000× *g* at 4 °C for 20 min and the supernatant was used to determine the O_2_^•−^ concentration according to Chaves et al. [22].

#### 2.11.4. Determining Antioxidant Enzyme Activities

The leaf tissue (0.1 g) was ground as described above and the fine powder was homogenized with 1 mL of solution containing 100 mM of potassium phosphate buffer (pH 7.8), 0.1 mM of EDTA, 1 mM of phenylmethyl–sulphonyl fluoride, and 0.5% (*w*/*v*) polyvinylpolypyrrolidone. The homogenate was centrifuged at 13,000× *g* for 15 min at 4 °C and the supernatant was used to determine the activities of ascorbate peroxidase (APX) (EC 1.11.1.11), catalase (CAT) (EC 1.11.1.6), superoxide dismutase (SOD) (EC 1.15.1.1), and glutathione reductase (GR) (EC 1.8.1.7) following the procedure of Debona et al. [23].

#### 2.11.5. Gene Expression Using Reverse Transcription Quantitative Real-Time PCR (RT-PCR)

The leaf tissue (0.1 g) was ground as described above and the fine powder was used to extract the RNA using TRIzol (Invitrogen, São Paulo, Brazil). Contamination by DNA was eliminated using RQ1 RNase-Free DNase (Promega, São Paulo, Brazil). The amount of RNA was measured in a Qubit fluorometer using a Qubit RNA HS assay kit (Invitrogen) and the RNA quality and integrity were verified by 1% agarose gel electrophoresis. Single-stranded cDNAs were synthesized via reverse transcription using 3 μg of total RNA with oligo(dT) primers in a final volume of 20 μL using the SuperScript First Strand Synthesis System for RT-PCR (Invitrogen). The qRT-PCR was performed on a Bio-Rad CFX Real Time Thermal Cycler using SYBR Green PCR Master Mix according to the recommendations of the manufacturer. All reactions were performed in duplicate and the relative expression values for each gene studied were calculated using the 2^−ΔΔCt^ method [24]. Expression analyses of genes encoding for indole-3-glycerol phosphate lyase (*IGL*), chalcone synthase (*CHS02*), pathogenesis-related protein 1 (*PR1*), linoleate 9S-lipoxygenase3 (*LOX3*), phenylalanine ammonia-lyase 3 (*PAL3*), endochitinase (*CHI*), and endo-1,3(4)-β-D-glucanase (*GLU*) were performed using specific primer sequences (Table S1). The expression of the non-ribosomal peptide synthetase gene from *B. maydis* (*Bm*) was quantified to confirm its presence in the leaf tissues of maize plants [25]. The gene encoding for cytosolic glyceraldehyde-3-phosphate dehydrogenase (*GAPDH*) was used as a reference for normalization [26].

### 2.12. Experimental Design and Statistical Analysis

For the in vitro assays, the experiment was arranged in a completely randomized design (CRD) with six treatments (control and five concentrations of IR stimulus). A total of ten replications were used for either the glass slide or Petri dish assays. Each replication corresponded to one glass slide or a Petri dish. A 2 × 2 factorial experiment was arranged in a CRD with four replications per evaluation time to assess the disease severity, as well to determine the foliar concentrations of Zn and N. The factors studied were plants sprayed with water (control) or with IR stimulus (named products) and non-inoculated or inoculated with *B. maydis* (named plant inoculation). Another 2 × 2 factorial experiment was arranged in a CRD with six replications per evaluation time, and the same factors mentioned above were used to evaluate the leaf gas exchange and Chl *a* fluorescence parameters, as well to quantify the foliar concentrations of pigments. The leaf samples for the biochemical assays and gene expression analysis were obtained from another 2 × 2 factorial experiment arranged in a CRD with five replications per evaluation time and the same factors mentioned above. Each experimental unit consisted of one plastic pot with two plants. All experiments were repeated once. The data from the variables and parameters were checked for normality and homogeneity of variance and subjected to an analysis of variance (ANOVA). The treatment means were compared using F or Tukey tests (*p* ≤ 0.05). Data from all variables and parameters obtained from the four treatments at 156 hai were used for the principal component analysis (PCA). A statistical analysis of all data obtained was carried out using the Minitab Statistical software version 22.1.0 (State College, PA, USA) [27].

## 3. Results

### 3.1. Analysis of Variance

The factor IR stimulus was significant for most of the variables and parameters studied, except for Y(NPQ), glucose, fructose, APX, and GR (Table S2). The factor plant inoculation (PI), sampling time (ST), and interactions for IR × PI, IR × ST, PI × ST, and IR × PI × ST were significant for most of the variables and parameters evaluated (Table S2).

### 3.2. In Vitro Assay

**Figure 1:**
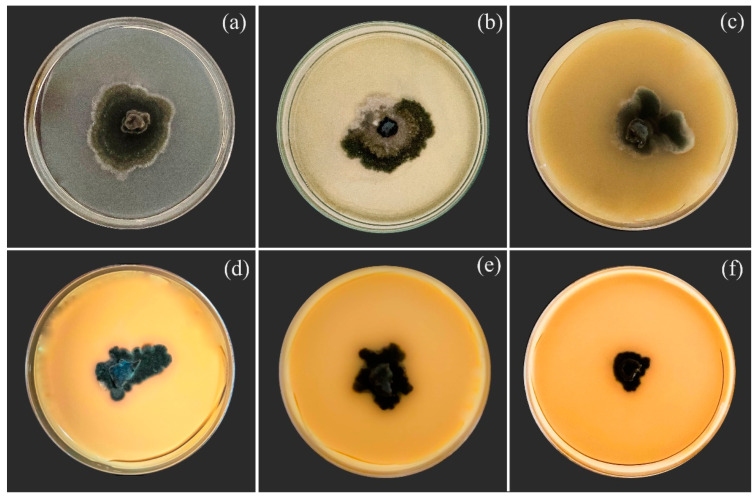

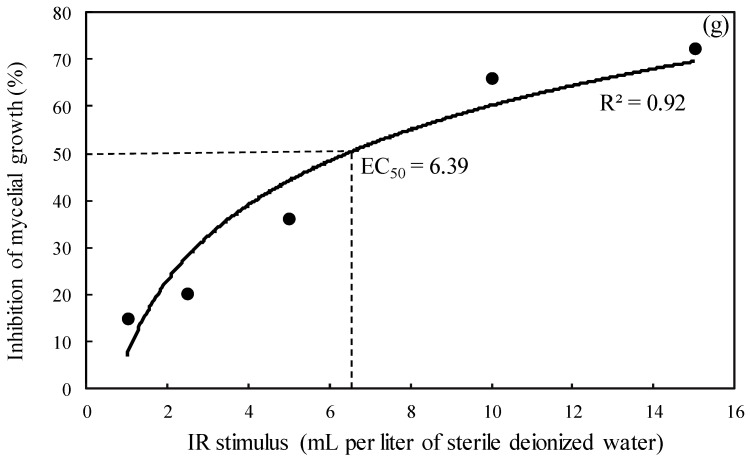
Mycelial growth of *Bipolaris maydis* in Petri dishes containing potato dextrose agar amended with 0 (**a**), 1 (**b**), 2.5 (**c**), 5 (**d**), 10 (**e**), and 15 (**f**) mL of induced resistance (IR) stimulus per liter of sterile deionized water. Effective concentration (EC_50_) of IR stimulus that inhibited 50% of the mycelial growth from *B. maydis* (**g**).

**Figure 2:**
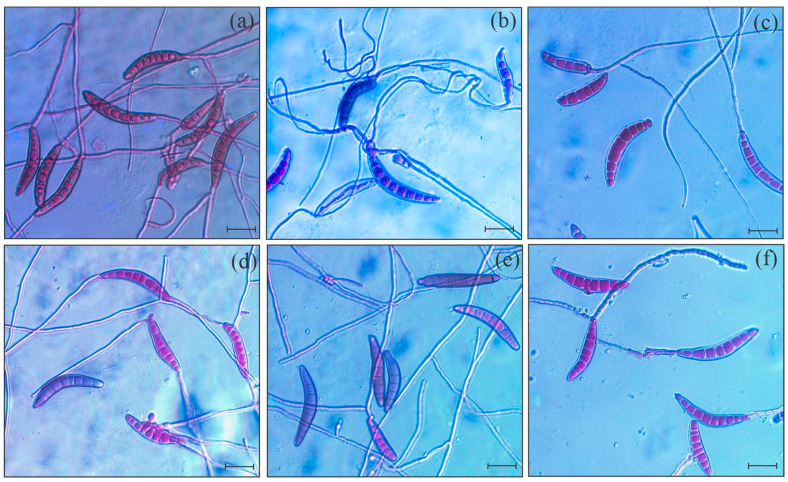
Visual aspect of the germination of conidia from *Bipolaris maydis*) previously exposed to 0 (**a**), 1 (**b**), 2.5 (**c**), 5 (**d**), 10 (**e**), and 15 (**f**) mL of induced resistance (IR) stimulus per liter of sterile deionized water. Bars = 10 µm.

The size and appearance of the fungal colonies were affected by the IR stimulus, with the rates increased from 1 to 15 mL/L (Figure 1a–f). The EC_50_ obtained for the IR stimulus was 6.4 mL/L (Figure 1g). In comparison to the control treatment (Figure 2a), the germinated conidia of *B. maydis* had thin and shorter germ tubes when exposed to IR stimulus rates ranging from 1 to 15 mL/L (Figure 2b–f). The conidium germination significantly decreased by 6, 9, 18, 31, and 46% for 1, 2.5, 5, 10, and 15 mL of IR stimulus/L, respectively, compared to the control treatment (Figure S1).

### 3.3. Foliar Concentrations of Zn and N

**Figure 3:**
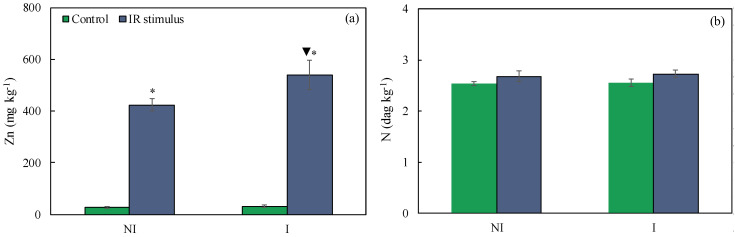
Foliar concentrations of zinc (Zn) (**a**) and nitrogen (N) (**b**) for maize plants non-inoculated or inoculated with *Bipolaris maydis* and sprayed with water (control) or with induced resistance (IR) stimulus. Means for NI and I treatments followed by an inverted triangle (▼) and for control and IR stimulus treatments followed by an asterisk (*) are significantly different according to the *F* test (*p* ≤ 0.05). Bars represent the standard error of the means.

The foliar Zn concentrations for the non-inoculated and IR-stimulus-sprayed plants and inoculated and IR-stimulus-sprayed plants significantly increased by 93 and 94%, respectively, compared to the non-inoculated and inoculated plants from the control treatment (Figure 3a). For the inoculated and IR-stimulus-sprayed plants, the foliar Zn concentration significantly increased by 27% compared to the non-inoculated and IR-stimulus-sprayed plants (Figure 3a). There was no significant difference for the foliar N concentrations between the IR stimulus and control treatments regardless of plant inoculation with *B. maydis* (Figure 3b).

### 3.4. Symptoms of MLB and Disease Severity

**Figure 4:**
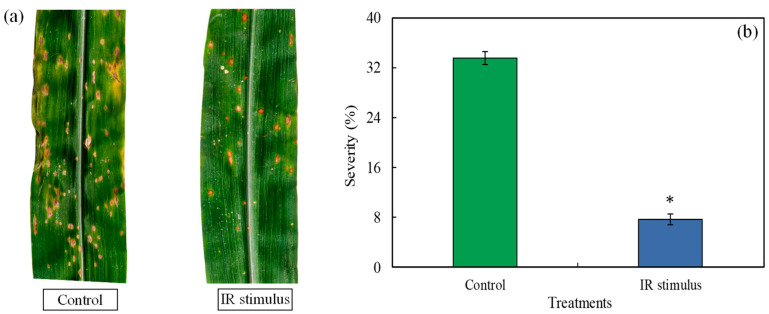
Symptoms of *Bipolaris* leaf spot (**a**) and disease severity (**b**) for maize plants sprayed with water (control) or with induced resistance (IR) stimulus. The asterisk (*) indicates statistical significance (*p* ≤ 0.05) between control and IR stimulus treatments (graph **b**) according to the *F* test. Bars in graph (**b**) represent the standard error of the means. Disease symptoms and severity were obtained at seven days after inoculation of plants with *Bipolaris maydis*.

Many necrotic and elliptical lesions developed in the leaves of the maize plants from the control treatment, while the lesions that formed in the leaves of the IR-stimulus-sprayed plants were of reduced size and less in number (Figure 4a). The MLB severity was significantly reduced by 76% for the IR-stimulus-sprayed plants compared to the plants from the control treatment (Figure 4b).

### 3.5. Leaf Gas Exchange Parameters

**Figure 5:**
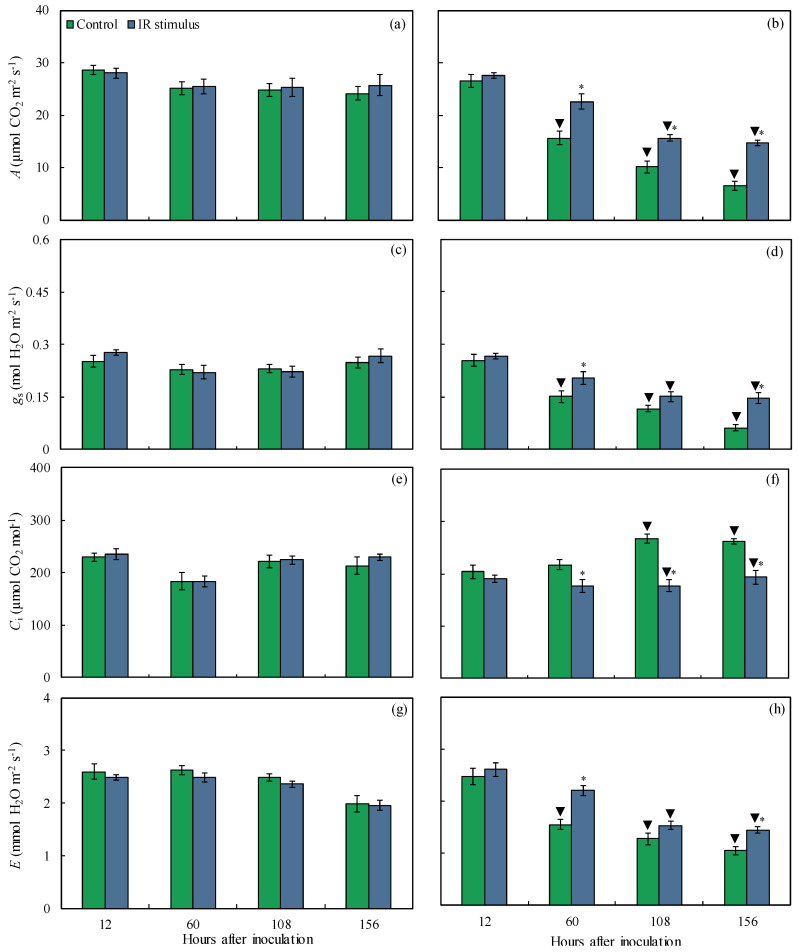
Leaf gas exchange parameters. Rates of net carbon assimilation (*A*) (**a**,**b**), stomatal conductance to water vapor (*g*_s_) (**c**,**d**), internal CO_2_ concentration (C_i_) (**e**,**f**), and transpiration (*E*) (**g**,**h**) determined on the leaves of maize plants non-inoculated (NI) (**a**,**c**,**e**,**g**) or inoculated (I) (**b**,**d**,**f**,**h**) with *Bipolaris maydis* and sprayed with water (control) or with induced resistance (IR) stimulus. Means for NI and I treatments followed by an inverted triangle (▼) and for control and IR stimulus treatments followed by an asterisk (*), at each evaluation time, are significantly different (*p* ≤ 0.05) according to the *F* test. Bars represent the standard error of the means.

For the non-inoculated plants, the *A*, *g*_s_, *C*_i_, and *E* values were not affected by the IR stimulus compared to the control treatment, regardless of the evaluation time (Figure 5a,c,e,g). For the inoculated and IR-stimulus-sprayed plants, the *A* (31, 35, and 56% at 60, 108, and 156 hai, respectively), *g*_s_ (26 and 57% at 60 and 156 hai, respectively), and *E* (30 and 27% at 60 and 156 hai, respectively) values were significantly higher while the *C*_i_ value (19, 34, and 26% at 60, 108, and 156 hai, respectively) was significantly lower compared to the inoculated and water-sprayed plants (Figure 5b,d,f,h). For the control treatment, the *A* (38–73% from 60 to 156 hai), *g*_s_ (34–75% from 60 to 156 hai), and *E* (41–48% from 60 to 156 hai) values were significantly lower while the *C*_i_ value (17 and 19% at 108 and 156 hai, respectively) was significantly higher for the inoculated compared to non-inoculated plants (Figure 5a–h and Figure 5e,f). The *A* (38 and 43%), *g*_s_ (32 and 46%), *C*_i_ (21 and 16%), and *E* (35 and 25%) values were significantly lower at 108 and 156 hai for the inoculated and IR-stimulus-sprayed plants compared to their non-inoculated counterparts (Figure 5a–h).

### 3.6. Imaging and Quantification of Chl a Fluorescence Parameters

**Figure 6:**
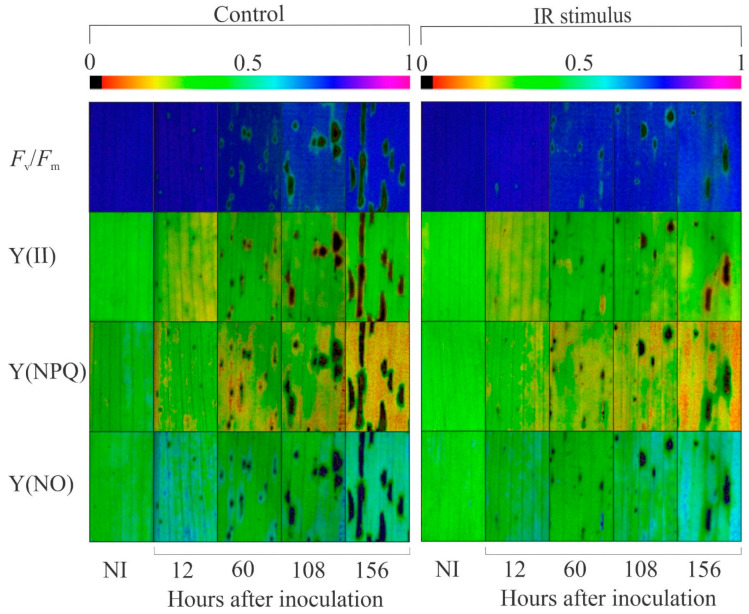
Images of chlorophyll *a* fluorescence parameters: maximum PSII quantum efficiency (*F*_v_/*F*_m_), photochemical yield (Y(II)), yield for dissipation by down-regulation (Y(NPQ)), and yield for non-regulated dissipation (Y(NO)) for leaves of maize plants that were non-inoculated (NI) or at different times after inoculation with *Bipolaris maydis* that were sprayed with water (control) or with induced resistance (IR) stimulus.

**Figure 7:**
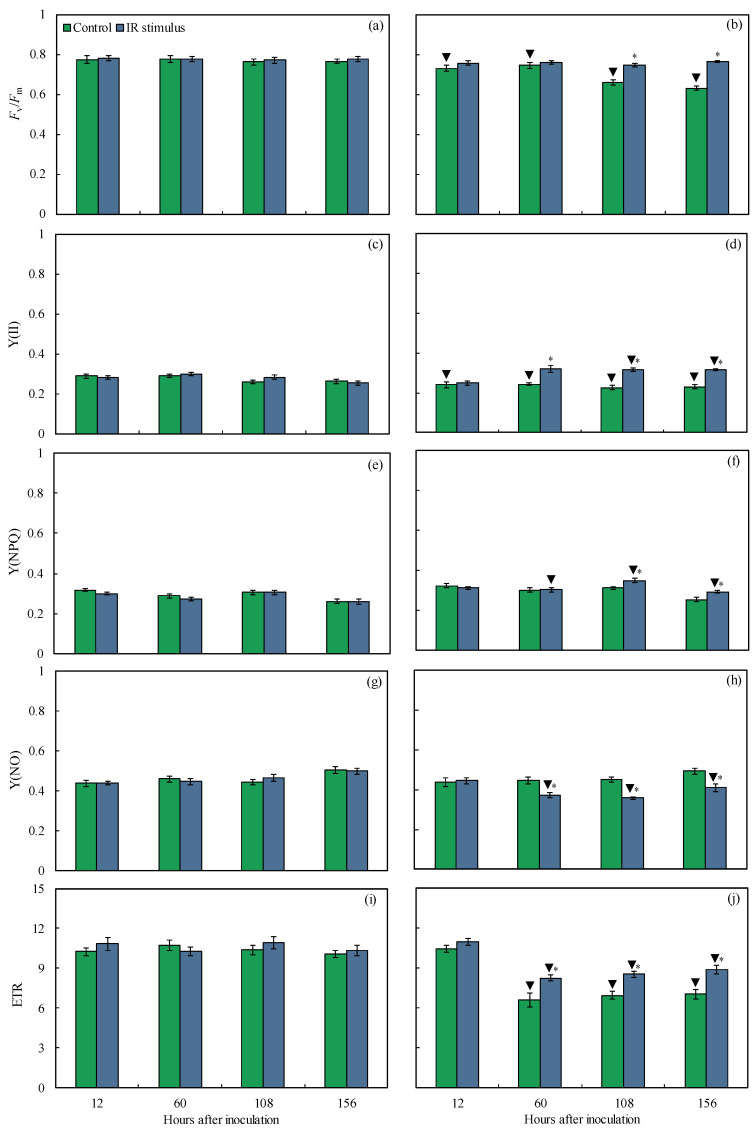
Quantification of chlorophyll *a* fluorescence parameters: maximum PSII quantum efficiency (*F*_v_/*F*_m_) (**a**,**b**), photochemical yield (Y(II)) (**c**,**d**), yield for dissipation by down-regulation (Y(NPQ)) (**e**,**f**), yield for non-regulated dissipation (Y(NO)) (**g**,**h**), and electron transport rate (ETR) (**i**,**j**) determined on leaves of maize plants that were non-inoculated (NI) (**a**,**c**,**e**,**g**,**i**) or inoculated (I) (**b**,**d**,**f**,**h**,**j**) with *Bipolaris maydis* and sprayed with water (control) or with induced resistance (IR) stimulus. Means for NI and I treatments followed by an inverted triangle (▼) and for control and IR stimulus treatments followed by an asterisk (*), at each evaluation time, are significantly different (*p* ≤ 0.05) according to the *F* test. Bars represent the standard error of the means.

Damage to the photosynthetic apparatus was noticed in the leaves of the inoculated plants from the control treatment compared to the leaves of the inoculated and IR-stimulus-sprayed plants based on the darker areas in the images for the *F*_v_/*F*_m_, Y(II), Y(NPQ), and Y(NO) parameters (Figure 6). For the non-inoculated plants, there was no significant difference between the control and IR stimulus treatments, regardless of the evaluation time (Figure 7a,c,e,g). For the inoculated plants, the *F*_v_/*F*_m_ (12 and 18% at 108 and 156 hai, respectively), Y(II) (24, 29, and 27% at 60, 108, and 156 hai, respectively), Y(NPQ) (11 and 14% at 108 and 156 hai, respectively), and ETR (20, 19, and 21% at 60, 108 and 156 hai, respectively) parameters were significantly higher, while the Y(NO) value (16–21% from 60 to 156 hai) was significantly lower for the IR-stimulus-sprayed plants compared to the plants from the control treatment (Figure 7b,d,f,h,j). For the control treatment, the *F*_v_/*F*_m_ (6–18% from 12 to 156 hai), Y(II) (13–17% from 12 to 156 hai), and ETR (30–38% from 60 to 156 hai) values were significantly lower for inoculated compared to the non-inoculated plants (Figure 7a–d,i,j). For the IR stimulus treatment, the Y(II) (11 and 20% at 108 and 156 hai, respectively) and Y(NPQ) (11–13% from 60–156 hai) values were significantly higher while the Y(NO) and ETR values were significantly lower for the inoculated plants compared to the non-inoculated plants from 60 to 156 hai (Figure 7c–j).

### 3.7. Photosynthetic Pigments

**Figure 8:**
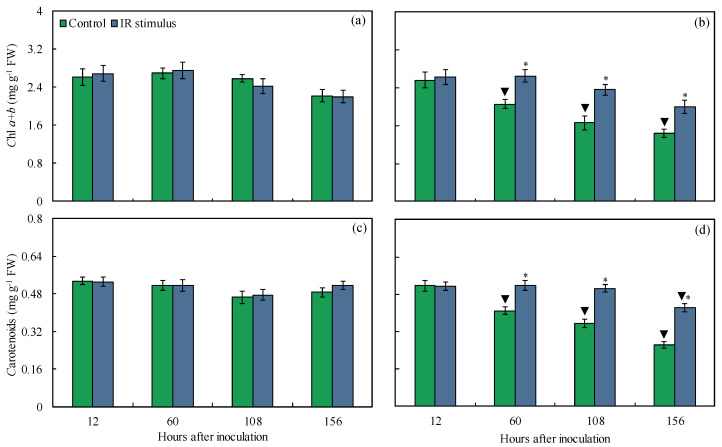
Concentrations of chlorophyll *a*+*b* (Chl *a+b*) (**a**,**b**) and carotenoids (**c**,**d**) determined on leaves of maize plants non-inoculated (NI) (**a**,**c**) or inoculated (I) (**b**,**d**) with *Bipolaris maydis* and sprayed with water (control) or with induced resistance (IR) stimulus. Means for NI and I treatments followed by an inverted triangle (▼) and for control and IR stimulus treatments followed by an asterisk (*), at each evaluation time, are significantly different (*p* ≤ 0.05) according to the *F* test. Bars represent the standard error of the means. FW = fresh weight.

The concentrations of Chl *a*+*b* and carotenoids for the non-inoculated plants were not affected by the IR stimulus compared to the control treatment, regardless of the evaluation time (Figure 8a,c). For the inoculated plants, the concentrations of Chl *a*+*b* (22–30%) and carotenoids (21–38%) were significantly higher for the IR-stimulus-sprayed plants compared to the plants from the control treatment from 60 to 156 hai (Figure 8b,d). For the control treatment, the concentrations of Chl *a*+*b* (24–36%) and carotenoids (21–46%) were significantly lower for the inoculated compared to non-inoculated plants (Figure 8a–d). For the IR stimulus treatment, the concentration of carotenoids was significantly reduced by 18% for the inoculated compared to non-inoculated plants at 156 hai (Figure 8a–d).

### 3.8. Carbohydrates

**Figure 9:**
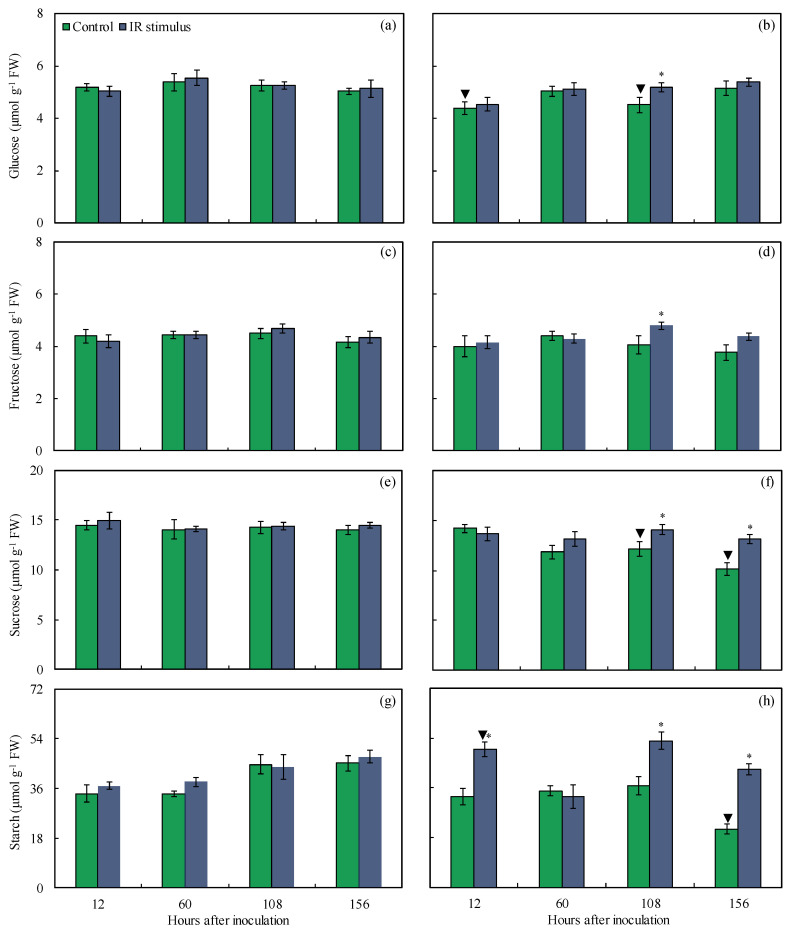
Concentrations of glucose (**a**,**b**), fructose (**c**,**d**), sucrose (**e**,**f**), and starch (**g**,**h**) determined on leaves of maize plants non-inoculated (NI) (**a**,**c**,**e**,**g**) or inoculated (I) (**b**,**d**,**f**,**h**) with *Bipolaris maydis* and sprayed with water (control) or with induced resistance (IR) stimulus. Means for NI and I treatments followed by an inverted triangle (▼) and for control and IR stimulus treatments followed by an asterisk (*), at each evaluation time, are significantly different (*p* ≤ 0.05) according to the *F* test. Bars represent the standard error of the means. FW = fresh weight.

For the non-inoculated plants, there was no significant difference between the control and IR stimulus treatments, regardless of the evaluation time (Figure 9a,c,e,g). For the inoculated and IR-stimulus-sprayed plants, the glucose (13% at 108 hai), fructose (15% at 108 hai), sucrose (14 and 22% at 108 and 156 hai, respectively), and starch (34, 31, and 51% at 12, 60, 156 hai, respectively) concentrations were significantly higher compared to the inoculated plants from the control treatment (Figure 9b,d,f,h). For the control treatment, the concentrations of glucose (17 and 14% at 12 and 108 hai, respectively), sucrose (15 and 27% at 108 and 156 hai, respectively), and starch (52% at 156 hai) were significantly lower for the inoculated compared to non-inoculated plants (Figure 9a,b,e–h). For the IR stimulus treatment, the concentration of starch significantly increased by 26% for the inoculated compared to non-inoculated plants at 12 hai (Figure 9g,h).

### 3.9. Histochemical Assays

**Figure 10:**
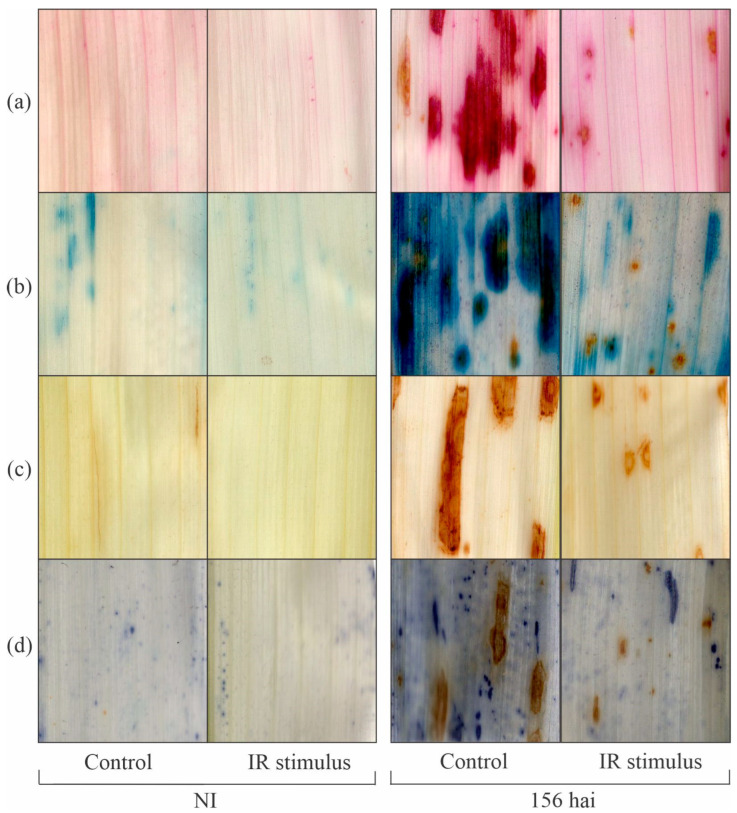
Histochemical detection of lipid peroxidation (**a**), membrane damage (**b**), hydrogen peroxide (**c**), and superoxide anion radical (**d**) on leaves of maize plants that were non-inoculated or at 156 h after inoculation (hai) with *Bipolaris maydis* that were previously sprayed with water (control) or with induced resistance (IR) stimulus.

No sign of cellular perturbation on the leaves from the non-inoculated and IR-stimulus-sprayed plants was noticed based on the absence of staining for lipid peroxidation, membrane damage, and depositions of H_2_O_2_ and O_2_^•−^ compared to the leaves of the non-inoculated plants from the control treatment (Figure 10a–d). The lipid peroxidation (pink color), membrane damage (blue color), and depositions of H_2_O_2_ and O_2_^•−^ (brown and blue colors, respectively) were less intense in the leaves of the inoculated and IR-stimulus-sprayed plants than on the leaves of the inoculated plants from the control treatment at 156 hai (Figure 10a–d).

### 3.10. Concentrations of MDA, H_2_O_2_, and O_2_^•−^

**Figure 11:**
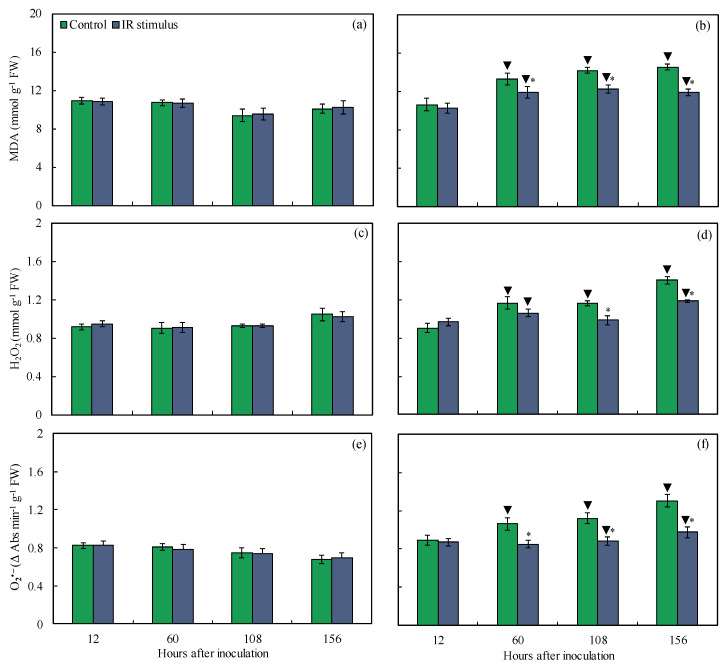
Concentrations of malondialdehyde (MDA) (**a**,**b**), hydrogen peroxide (H_2_O_2_) (**c**,**d**), and superoxide anion radical (O_2_^•−^) (**e**,**f**) determined on leaves of maize plants that were non-inoculated (NI) (**a**,**c**,**e**) or inoculated (I) (**b**,**d**,**f**) with *Bipolaris maydis* and sprayed with water (control) or with induced resistance (IR) stimulus. Means for NI and I treatments followed by an inverted triangle (▼) and for control and IR stimulus treatments followed by an asterisk (*), at each evaluation time, are significantly different (*p* ≤ 0.05) according to the *F* test. Bars represent the standard error of the means. FW = fresh weight.

The concentrations of MDA, H_2_O_2_, and O_2_^•−^ for the non-inoculated plants were not affected by the IR stimulus compared to the control treatment, regardless of the evaluation time (Figure 11a,c,e). For the inoculated plants, the MDA (10–18% from 60 to 156 hai), H_2_O_2_ (15 and 16% at 108 and 156 hai, respectively), and O_2_^•−^ (20–25% from 60 to 156 hai) concentrations were significantly lower for the IR-stimulus-sprayed plants compared to the plants from the control treatment (Figure 11b,d,f). For the control treatment, the MDA (19–34%), H_2_O_2_ (20–26%), and O_2_^•−^ (24–48%) concentrations were significantly higher for the inoculated plants compared to the non-inoculated plants from 60 to 156 hai. For the IR stimulus treatment, the MDA (10, 22, and 14% at 60, 108, and 156 hai, respectively), H_2_O_2_ (14 and 15% at 60 and 156 hai, respectively), and O_2_^•−^ (16 and 28% at 108 and 156 hai, respectively) concentrations were significantly higher for the inoculated compared to the non-inoculated plants (Figure 11a–f).

### 3.11. Antioxidant Enzymes

**Figure 12:**
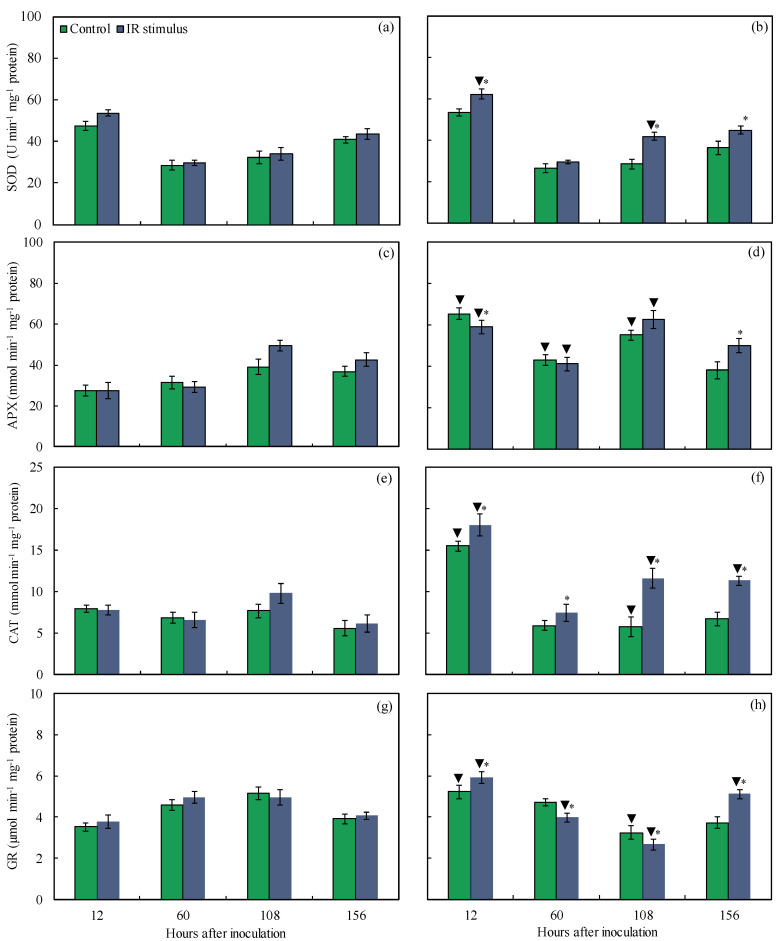
Activities of superoxide dismutase (SOD) (**a**,**b**), ascorbate peroxidase (APX) (**c**,**d**), catalase (CAT) (**e**,**f**), and glutathione reductase (GR) (**g**,**h**) determined on leaves of maize plants that were non-inoculated (NI) (**a**,**c**,**e**,**g**) or inoculated (I) (**b**,**d**,**f**,**h**) with *Bipolaris maydis* and sprayed with water (control) or with induced resistance (IR) stimulus. Means for NI and I treatments followed by an inverted triangle (▼) and for control and IR stimulus treatments followed by an asterisk (*), at each evaluation time, are significantly different (*p* ≤ 0.05) according to the *F* test. Bars represent the standard error of the means.

For the non-inoculated plants, there was no significant difference between the control and IR stimulus treatments, regardless of the evaluation time (Figure 12a,c,e,g). For the inoculated plants, the SOD (14, 11, and 19% at 12, 108, and 156 hai, respectively), APX (33% at 156 hai), CAT (14, 50, and 41% at 12, 108, and 156 hai, respectively), and GR (12 and 27% at 12 and 156 hai, respectively) activities were significantly higher for the IR-stimulus-sprayed plants compared to the plants from the control treatment (Figure 12b,d,f,h). The activities of APX (10% at 12 hai) and GR (16 and 18% at 60 and 108 hai, respectively) were significantly lower for the inoculated and IR-stimulus-sprayed plants compared to the inoculated plants from the control treatment (Figure 12d,h). For the control treatment, the activities of APX (58, 27, and 29% at 12, 60, and 108 hai, respectively), as well as those of CAT and GR (49 and 33% at 12 hai, respectively), were significantly higher for the inoculated compared to the non-inoculated plants (Figure 12c–h). For the IR stimulus treatment, the SOD (14% at 12 hai), APX (53, 29, and 21% at 12, 60, and 108 hai, respectively), CAT (49, 16, and 46% at 12, 108, and 156 hai, respectively), and GR (36 and 21% at 12 and 156 hai, respectively) activities were significantly higher for the inoculated compared to the non-inoculated plants (Figure 12a–h).

### 3.12. Gene Expression

**Figure 13:**
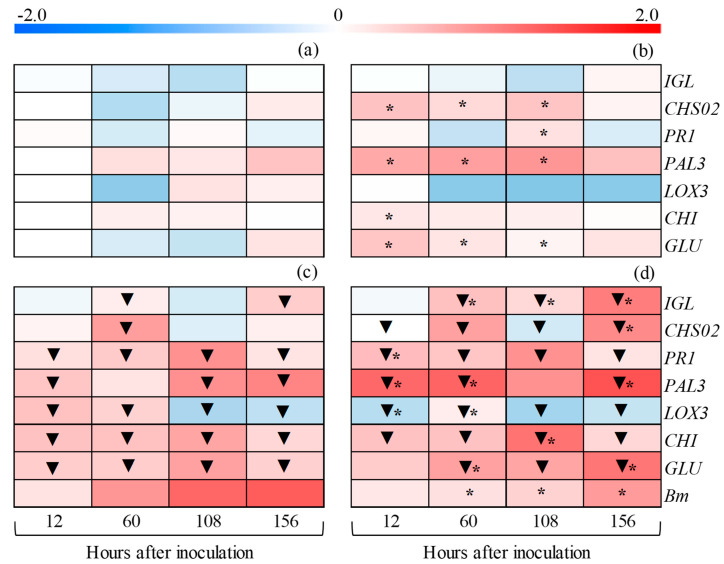
Expression profile of genes determined on leaves of maize plants that were non-inoculated (NI) (**a**,**b**) or inoculated (I) (**c**,**d**) with *Bipolaris maydis* and sprayed with water (control) (**a**,**c**) or with induced resistance (IR) stimulus (**b**,**d**). Color cells represent the relative transcript levels ranging from blue (−2.0) to red (2.0). The amplification of cytosolic glyceraldehyde-3-phosphate dehydrogenase (*GAPDH*) from maize was used as an internal control for data normalization. Fold changes for each gene expression were calculated based on the transcript level obtained for leaves from NI plants of control treatment at 12 h after inoculation (hai), except for the *Bm* gene from *B. maydis*. For *Bm*, the transcript level obtained for leaves from I plants of control treatment at 12 hai was used in the calculation. For each leaf sample, four biological replications were used with three technical replicates each. Means for NI and I treatments (comparisons between (**a**,**c**) and (**b**,**d**)) followed by an inverted triangle (▼) and for control and IR stimulus treatments (comparisons between (**a**,**b**) and (**c**,**d**)) followed by an asterisk (*), at each evaluation time, are significantly different (*p* ≤ 0.05) according to the *F* test.

Comparing non-inoculated vs. inoculated plants for control and IR stimulus treatments: For the control treatment, the expression levels of *PR1*, *PAL3*, *LOX3*, *CHI*, and *GLU* at 12 hai; *IGL*, *CHS02*, *PR1*, *LOX3*, *CHI*, and *GLU* at 60 hai; *PR1*, *PAL3*, *CHI*, and *GLU* at 108 hai; and *IGL*, *PR1*, *PAL3*, *CHI*, and *GLU* at 156 hai were significantly higher for the inoculated plants compared to the non-inoculated plants. The expression levels of *LOX3* at 108 and 156 hai were significantly reduced for the inoculated plants compared to the non-inoculated plants of the control treatment (Figure 13a,c). The expression levels of *PR1*, *PAL3*, and *CHI* at 12 hai; *IGL*, *CHS02*, *PR1*, *PAL3*, *LOX3*, *CHI*, and *GLU* at 60 hai; *IGL*, *PR1*, *LOX3*, *CHI*, and *GLU* at 108 hai; and *IGL*, *PR1*, *PAL3*, *LOX3*, *CHI*, and *GLU* at 156 hai were significantly higher for the inoculated plants compared to the non-inoculated plants of the IR stimulus treatment. For the IR stimulus treatment, the expression levels of *LOX3* at 12 hai and *CHS02* at 12 and 108 hai were significantly reduced for the inoculated plants compared to the non-inoculated plants (Figure 13b,d).

Comparing IR stimulus and control treatments for non-inoculated and inoculated plants: For the non-inoculated plants, the expression levels of *CHS02*, *PAL3*, *CHI*, and *GLU* at 12 hai; *CHS02*, *PAL3*, and *GLU* at 60 hai; and *CHS02*, *PR1*, *PAL3*, and *GLU* at 108 hai were significantly up-regulated for the IR stimulus treatment compared to the control treatment (Figure 13a,b). For the inoculated plants, the expression levels of *PR1* and *PAL3* at 12 hai; *IGL*, *PAL3*, and *GLU* at 60 hai; *IGL* and *CHI* at 108 hai; and *IGL*, *CHS02*, *PAL3*, and *GLU* at 156 hai were significantly up-regulated for the IR stimulus treatment compared to the control treatment (Figure 13c,d). The expression levels of *LOX3* at 12 and 60 hai and *Bm* from 60 to 156 hai were significantly reduced for the IR stimulus treatment compared to the control treatment (Figure 13c,d).

### 3.13. PCA

**Figure 14:**
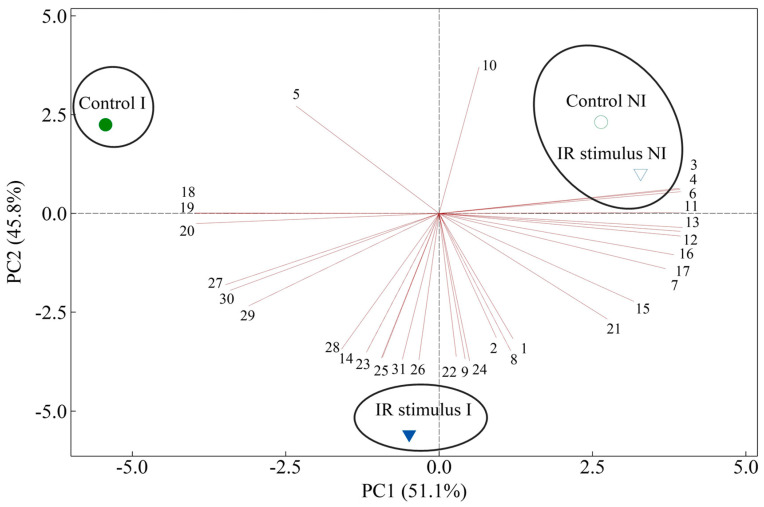
Score and loading plots of the principal component analysis (PCA) for variables and parameters determined on leaves of maize plants that were non-inoculated (NI) or inoculated (I) with *Bipolaris maydis* and sprayed with water (control) or with induced resistance (IR) stimulus. Numbers in the leading plot are for foliar concentrations of Zn and N (1 and 2, respectively), leaf gas exchange (3, 4, 5, and 6 respectively correspond to *A*, *g*_s_, *C*_i_, and *E*) chlorophyll *a* fluorescence (7, 8, 9, 10, and 11 respectively correspond to *F*_v_/*F*_m_, Y(II), Y(NPQ), Y(NO), and ETR); concentrations of photosynthetic pigments (12 and 13 respectively correspond to Chl *a*+*b* and Car), carbohydrates (14, 15, 16, and 17 respectively correspond to glucose, fructose, sucrose, and starch), and metabolites (18, 19, and 20 respectively correspond to MDA, H_2_O_2_, and O_2_^•−^); activities of antioxidant enzymes (21, 22, 23, and 24 respectively correspond to SOD, APX, CAT, and GR); and gene expression (25, 26, 27, 28, 29, 30, and 31 respectively correspond to *IGL*, *CHS02*, *PR1*, *PAL*, *LOX3*, *CHI*, and *GLU*). Groups were generated from a cluster analysis with complete linkage and a Pearson distance. Data from variables and parameters used in the PCA analysis were obtained from NI and I plants at 156 h after inoculation.

According to the cluster analysis with complete linkage and a Pearson distance, three clusters were generated: inoculated plants from the control treatment, inoculated plants from the IR stimulus treatment, and non-inoculated plants from the control and IR stimulus treatments (Figure 14). One principal component (PC) explained most of data variation (PC1 = 51.1% and PC2 = 45.8%) (Figure 14). PC1 indicated negative scores for *C*_i_, glucose, MDA, H_2_O_2_, O_2_^●−^, CAT, *IGL*, *CHS02*, *PR1*, *PAL3*, *LOX3*, *CHI*, and *GLU,* while positive scores were obtained for Zn, N, *A*, *g*_s_, *E*, *F*_v_/*F*_m_, Y(II), Y(NPQ), Y(NO), ETR, Chl *a*+*b*, carotenoids, fructose, sucrose, starch, SOD, APX, and GR. PC2 was characterized by negative scores for Zn, N, *F*_v_/*F*_m_, Y(II), Y(NPQ), Chl *a*+*b*, carotenoids, glucose, fructose, sucrose, starch, O_2_^●−^, SOD, AOX, CAT, GR, *IGL*, *CHS02*, *PR1*, *PAL3*, *LOX3*, *CHI*, and *GLU,* while positive scores were obtained for *A*, *g*_s_, *C*_i_, *E*, Y(NO), ETR, MDA, and H_2_O_2_ (Figure 14).

## 4. Discussion

The use of IR stimuli represents a sustainable alternative to complement the currently recommended control methods for destructive diseases affecting profitable crops such as maize [6,7]. In the present study, the MLB symptoms and fungal colonization on leaf tissues were reduced for the IR-stimulus-sprayed plants. The potential of the IR stimulus to trigger maize defense reactions against infection by *B. maydis* was clearly confirmed at the physiological, biochemical, and molecular levels. Interestingly, the IR stimulus negatively affected the mycelial growth of *B. maydis* and conidium germination in vitro, possibly due to osmotic stress, ion imbalance, and changes in membrane integrity. Some IR stimuli such as different formulations of phosphites, oxalic acid, saccharin, and a copper-polyphenolic compound were capable of exerting an antimicrobial effect against different pathogens, mainly through the rupture of the hyphae cell wall, which resulted in greater production of electrolyte leakage [5,11,15,22,28].

In the present study, the IR-stimulus-sprayed plants infected by *B. maydis* displayed higher Zn foliar concentrations in contrast to the non-infected ones. The reduced foliar symptoms of MLB for the IR-stimulus-sprayed plants can be accounted to higher Zn concentrations. One cannot disregard in this context the plausible co-participation of the polyphenolics contained in the IR stimulus for reducing the MLV symptoms. The Zn is a catalytic and structural protein cofactor of certain enzymes such as superoxide dismutase and alcohol dehydrogenase, in addition to its key structural functions in the protein domains of metallothionein, which act as antioxidants against reactive oxygen species (ROS) produced due to infection by pathogens [12]. The Zn finger proteins (Znf), which contain one or more Zn ions to stabilize their structure, are involved in the regulation of plant defense reactions against pathogen infection [12]. A meta-analysis study to understand the role of Znf in proteins of resistance (R) genes found 70 proteins related to the resistance of various crops against different diseases, and among them 37% contained Znf domains [12]. The Zn increased the resistance to and inhibited the mycelial growth of *Curvularia lunata*, *Alternaria grandis*, and *Fusarium solani* on maize, potato, and wheat, respectively [12,13].

The limitations imposed by the infection of pathogens from different lifestyles on photosynthesis are associated with lower synthesis and translocation rates of photoasimilates, as well as altered transpiration on stomata [21]. Changes in host metabolism can be monitored by the chlorophyll fluorescence kinetics linked with the outcome of the gas exchange parameter measurements [29,30]. Particularly in maize, leaf infection by *B. maydis* seriously compromised photosynthesis, as indicated by changes in leaf gas exchange (lower *A*, *g*_s_, and *E* values) and Chl *a* fluorescence (lower *F*_v_/*F*_m_, Y(II), Y(NO), and ETR values) parameters associated with great reductions in the pool of photosynthetic pigments [4,31]. In the present study, the harmful effect caused by an infection of *B. maydis* on the photosynthesis of maize plants was alleviated by the IR stimulus. The higher *A*, *g*_s_, and *E* values obtained for diseased leaves of IR-stimulus-sprayed plants reflected their better physiological status due to the preservation of the stomatal function and reductions in biochemical and dysfunctional limitations. Considering the photosynthetic apparatus, the diseased leaves of the IR-stimulus-sprayed plants displayed smooth alterations in their photochemical performance based on having the greatest values for *F*_v_/*F*_m_, Y(II), and Y(NPQ). In the present study, there was a balance between ETR and *A*, indicating the effect of the flow of electrons and the rate of CO_2_ assimilation during the photosynthetic process on the infected leaves of the IR-stimulus-sprayed plants. According to Klughammer and Schreiber [30], Y(NO) indicates the fraction of energy that is dissipated through unregulated extinction processes (e.g., heat and fluorescence) due to closed reaction centers in the PSII at saturated light intensity. Interestingly, the Y(NO) values were lower for the infected and IR-stimulus-sprayed plants compared to the infected and water-sprayed plants. This finding may reflect less photodamage on leaf tissues due to a decrease in the amount of energy dissipated through the non-extinction of energy regulated at the PSII level. Similar findings were found for common bean plants sprayed with oxalic acid and infected by *Sclerotinia sclerotiorum* [15] and soybean plants sprayed with phosphite combined with free amino acids and infected by *Phakopsora pachyrhizi* [18]. The reduction in MLS symptoms in the leaves of the IR-stimulus-sprayed plants was associated with a higher concentration of photosynthetic pigments (Chl *a*+*b* and carotenoids), indicating greater preservation of their photosynthetic apparatus and an efficient use of light energy for carbon fixation. Different IR stimuli attenuated the stress imposed by the infection of fungal pathogens on the photosynthetic capacity of their hosts due to greater pools of chlorophylls and carotenoids [10,11,15,21].

The response of plants against infection by pathogens occurs through the activation of different sets of defense reactions that demand an abundant and constant supply of energy derived mainly from carbohydrate metabolism [32]. In general, the infected and water-sprayed plants showed reduced pools of sugars and starch compared to the infected and IR-stimulus-sprayed plants. The down-regulation of genes encoding for photosynthetic proteins associated with PSI and PSII reaction centers, ATP synthase, RuBisCo activase, and phosphoribulose kinase contributed to lowering the foliar concentrations of sugars and starch in pathogen-challenged plants [32]. The reduction in the foliar pool of sucrose was greater in comparison to hexose, fructose, and glucose for tomato plants infected by *Botrytis cinerea* due to the negative expression of photosynthetic-related genes and impaired photosynthesis [33]. Along with carbon depletion due to reduced photosynthesis, fungal pathogens (*Botryosphaeria dothidea* and *Valsa sordida*) causing canker symptoms affected the distribution of carbohydrates in the stems of poplar plants [34]. The foliar concentrations of glucose, fructose (at 108 hai), and sucrose (at 108 and 156 hai) for infected and IR-stimulus-sprayed plants were kept higher compared to infected and water-sprayed plants. The starch concentration, the main reserve of carbon in plant tissues, in infected leaves of IR-stimulus-sprayed plants was higher. In various host–fungal pathogen interactions, the concentrations of soluble sugars and starch allow the carbon skeletons to act as signals for the functioning of different metabolic pathways responsible for the synthesis of diverse defense-related metabolites [35]. Higher concentrations of sugars at the infection sites of *Magnaporthe oryzae* in rice leaves played an important role in constitutive and induced chemical defense [35]. An increase in the concentration of soluble sugars seemed to be a determining factor in the defense response of tomato plants sprayed with a phosphite combined with free amino acids against septoria leaf spot [10]. Taken together, these findings suggest a possible increase in the flux of carbon in IR-stimulus-sprayed plants for increased resistance against infection by *B. maydis*.

The intense cellular damage caused by infection of necrotrophic and hemibiotrophic pathogens in the tissues of their hosts causes the excessive production of ROS and the activation of an enzymatic antioxidant system takes place for reduced lipid peroxidation [29]. In the present study, discrete depositions of H_2_O_2_ and O_2_^●−^ on leaf tissues and their lower concentrations associated with less membrane damage and lipid peroxidation (lower MDA concentration) were noticed for IR-stimulus-sprayed plants as a result of reduced MLB symptoms. In general, the higher SOD, CAT, and GR activities for IR-stimulus-sprayed plants attenuated the excessive production of H_2_O_2_ and O_2_^●−^, and consequently lowered the pool of MDA during infection by *B. maydis*. Interestingly, the great SOD activity during the foliar infection by *B. maydis* for the IR-stimulus-sprayed plants helped to catalyze the dismutation of H_2_O_2_ and O_2_^●−^. In this scenario, the CAT activity played the pivotal role in the elimination of H_2_O_2_ rather than the APX activity, which seemed to be higher at an advance stage of infection by *B. maydis*. Neither the APX or GR activity increased in the leaves of soybean plants sprayed with a copper polyphenolic compound and infected by *P. pachyrhizi,* possibly linked to the lower production of singlet oxygen and hydroxyl radicals [11]. Plants such as common bean, rice, soybean, tomato, and wheat exposed to different IR stimuli (e.g., picolinic acid, glutamate, phosphites, and a copper polyphenolic compound) developed a more robust antioxidant machinery that involved great APX, CAT, GR, and SOD activity levels to interfere with the infection by pathogens of different lifestyles [10,11,15,21,29]. It is important to point out that more efficient defense reactions, along with a robust antioxidant metabolism gained by the IR-stimulus-sprayed plants, allowed them to respond against infection by *B. maydis* more efficiently at the infection sites.

Interestingly, most of the genes studied in the present study were up-regulated for the IR-stimulus-sprayed plants compared to the water-sprayed plants. This finding indicates the potential of this IR stimulus to elicit maize defense responses in the absence of infection by *B. maydis*. Notably, the pattern of gene expression for the infected plants was more evident upon their exposition to the IR stimulus. Interestingly, *IGL* (from 60 to 156 hai), *CHS02* (at 156 hai), *PR1* (at 12 hai), *PAL3* (at 12, 60, and 156 hai), *CHI* (at 108 hai), and *GLU* (at 60 and 156 hai) were strongly up-regulated in maize leaves of IR-stimulus-sprayed plants facing infection by *B. maydis,* highlighting their contribution to an increased resistance against MLB. Plants exogenously exposed to different IR stimuli encountered profound changes at the physiological, transcriptional, and metabolic levels to have their defense capacity boosted against infection by pathogens [7,8]. Particularly in maize, different IR stimuli were capable of activating the expression of genes involved in different defense-related pathways in response to infection by pathogens [7,8,36]. The enzymatic roles of indole-glycerolphosphate lyases (a maize enzyme catalyzing the conversion of indole-3-glycerol phosphate to indole, which is subsequently converted into the benzoxazinoid secondary metabolites (DIBOA [2,4-di-hidroxi-2H-1,4-benzoxazin-3(4H)-ona] and its derivative methoxy DIMBOA [2,4-dihidroxi-7-metoxi-2H-1,4-benzoxazin-3(4H)-ona])) originated from *IGL* expression were similar to those enzymes coded by *Bx1* (benzoxazin 1) [37]. The contribution of benzoxazinoids is not only limited to their biocidal properties but also their role as regulatory signals to activate host defense responses against infections by *B. maydis* and *Exserohilum turcicum* in maize [38]. The importance of flavonoids and isoflavonoids for plant resistance against diseases is well recognized, and *CHS02* expression plays a key role in the regulation of their biosynthesis [39]. Soybean plants sprayed with a copper polyphenolic compound showed up-regulation of *CHIB1,* indicating the biosynthesis of flavonoids in response to infection by *P. pachyrhizi* [11]. The *PR1* was up-regulated only at 12 hai in infected maize leaves of IR-stimulus-sprayed plants. Manghwar et al. [40] reported the up-regulation of *PR1* in leaves infected by *Bipolaris sorokiniana,* highlighting its role in the increased resistance of maize plants. In tomato leaves sprayed with a phosphite combined with free amino acids and infected by *S. lycopersici*, *PR1b1* was up-regulated [10].

For IR-stimulus-sprayed plants, the phenylpropanoid pathway was shown to be important for their increased resistance against MLB, considering the up-regulation of *PAL3* at 12, 60, and 156 hai. PAL converts the aromatic amino acid phenylalanine to *trans*-cinnamic acid, from which a plethora of phenolics, flavonoids, and phytoalexins are originated, along with lignin production [41]. Maize resistance against infection by *B. maydis* was shown to be dependent on higher PAL activity [42]. In the present study, the up-regulation of *CHI* and *GLU* at 108 and 156 hai, respectively, for infected and IR-stimulus-sprayed plants was linked to their increased resistance against MLB. In maize plants, the expression of classes I and II of chitinase genes belonging to the PR-4 family contributed to their resistance against infection by *Fusarium moniliforme* [43]. The expression of *Chit2* in maize calluses affected their colonization by *Fusarium graminearum* [44]. Maize genotypes resistant to infection by *Fusarium verticillioides* exhibited great *β*-1,3-glucanase activity [45]. The resistance of wheat plants against infection by *Fusarium graminearum* was linked to great expression levels of *TaPR3* and *TaGlu2,* which encode for chitinase and *β*-1,3-glucanase, respectively [46]. In the present study, lower *LOX3* expression occurred for the infected and IR-stimulus-sprayed plants at 12 and 60 hai. In plant tissues infected by pathogens, especially necrotrophics, the lipoxygenases catalyze the oxidation of polyunsaturated fatty acids released by ROS-induced lipid peroxidation to produce oxylipins that will be enzymatically metabolized into traumatin and jasmonates [47]. Interestingly, the down-regulation of *LOX3* for the infected and IR-stimulus-sprayed plants may be attributed to the lower production levels of MDA and ROS in the smaller lesions originated from the infection of *B. maydis*.

In conclusion, the zinc polyphenolic compound showed potential to increase the maize resistance against MLB, considering collectively the physiological, biochemical, and molecular evidence reported in the present study. Based on the PCA analysis, the infected leaves of maize plants responded differently to the water and IR stimulus treatments. For the IR-stimulus-sprayed plants, in particular, a set of well-portrayed mechanisms such as a more preserved photosynthetic apparatus, the expression of genes involved in the host defense reactions, and a more robust antioxidant metabolism was of extreme relevance in impairing the infection process of *B. maydis*. In contrast, the physiological (leaf gas exchange and Chl *a* fluorescence parameters linked to the pool of photosynthetic pigments) and biochemical (concentrations of carbohydrates, MDA, H_2_O_2_, and O_2_^•^) responses of the water- and IR-stimulus-sprayed plants were quite similar in the absence of fungal infection. This finding indicates that the IR stimulus was able to modulate the metabolism of maize plants only after inoculation with *B. maydis*. It is tempting to assume that using this IR stimulus, combined with well-known control strategies, could become a promising alternative for MLB management in field conditions towards more sustainable agriculture. This option will definitively help to slow the epidemic rate of MLB, as well as to reduce the negative impacts imposed by the abusive use of fungicides on both human health and the environment.

## Data Availability

Data will be made available on reasonable request.

## Supplementary Materials

The following supporting information can be downloaded at: https://www.mdpi.com/article/10.3390/plants14010077/s1, Figure S1: Germination of conidia from *Bipolaris maydis* in Petri dishes containing potato-dextrose-agar medium non-amended (control) or amended with different rates of IR stimulus. Means from each treatment followed by different letters are significantly different (*p* ≤ 0.05) according to Tukey’s test. Bars represent the standard error of the means; Table S1: Genes and the primer sequences analyzed in the leaves of maize plants non-inoculated or inoculated with *Bipolaris maydis* and sprayed with water (control) or with induced resistance (IR) stimulus by using real-time quantitative reverse transcription PCR; Table S2: Analysis of variance for the effects of products (P), plant inoculation (PI), sampling time (ST), and their interactions on conidia germination (CG), foliar concentrations of zinc (Zn) and nitrogen (N), disease severity (Sev), leaf gas exchange parameters [rate of net CO_2_ assimilation (*A*), stomatal conductance to water vapour (*g*_s_), internal CO_2_ concentration (*C*_i_), and transpiration rate (*E*)], chlorophyll *a* fluorescence parameters [maximum PSII quantum efficiency (*F*_v_/*F*_m_), photochemical yield (Y(II)), yield for dissipation by down-regulation (Y(NPQ)), yield for non-regulated dissipation (Y(NO)), and electron transport rate (ETR)], concentrations of photosynthetic pigments (Chl *a*+*b* and carotenoids), carbohydrates (glucose, fructose, sucrose, and starch), malondialdehyde (MDA), hydrogen peroxide (H_2_O_2_), and superoxide anion radical (O_2_^•−^), activities of antioxidant enzymes [superoxide dismutase (SOD), ascorbate peroxidase (APX), catalase (CAT), and glutathione reductase (GR)] as well as expression of genes encoding for indole-3-glycerol phosphate lyase (*IGL*), chalcone synthase (*CHS02*), pathogenesis-related protein 1 (*PR1*), linoleate 9S-lipoxygenase3 (*LOX3*), phenylalanine ammonia-lyase 3 (*PAL3*), endochitinase (*CHI*), endo-1,3(4)-β-D-glucanase (*GLU*), and non-ribosomal peptide synthetase from *Bipolaris maydis* (*Bm*).
