# Supplementary material for: A Zinc Polyphenolic Compound Increases Maize Resistance Against Infection by Bipolaris maydis
Source: Plants (Basel). 2024 Dec 30;14(1):77. doi: 10.3390/plants14010077 (PMC11723224; doi:10.3390/plants14010077)
Supplement: Supplementary file 1 [file plants-14-00077-s001.zip › plants-3362832-supplementary.pdf]

Table S1

Genes	GenBank Id.	Primer sense 5'-3'	Primer antisense 5'-3'
IGL	NM_001301469.1	GCCTCATAGTTCCCGACCTC	GAATCCTCGTGAAGCTCGTG
CHS02	NM_001155550.1	TCACCGACCTCAAGGAGAAGTT	TGTACCGCTTCCGGATCATC
PR1	U82200.1	CCTACGGCGAGAACCTCTT	TCGTAGTACTGCTTCTCGGACA
PAL3	XM_020537583.3	AAGGTGTTCGTCGGCATC	TCCCACTCCTTGAGGCACT
LOX3	NM_001112045.1	CGCCAACTCCTGGGTCTAC	TCTGGCTTGGCAGGTACG
CHI	NM_001165432.1	GGTGCGAACGTGGCTAAT	CCGGGTGTAGAAGTTCTTGC
GLU	NM_001316316.1	CAGACCGGTCCATCCACGG	AGTACCCTGCCTTTGCAACCT
Bm	MN783607	TCTCGACAAGCAAATCAAAC	AGATGATTGCAGTGGTGTTG
GAPDH	NM_001111943.1	AAGCCGGTCACCGTCTTT	CATCTTTGCTTGGGGCAGA

Table S2

Variables/ Parameters	*F* values						
	P	PI	ST	P × PI	P × ST	PI × ST	P × PI × ST
CG	**257.11**	-	-	-	-	-	-
Zn	**538.14**	**15.23**	-	13.67	-	-	-
N	**5.5**	0.21	-	0.04	-	-	-
Sev	**356.82**	-	-	-	-	-	-
A	**23.68**	**195.83**	**53.64**	**16.92**	2.69	**24.10**	0.96
g_s	**12.91**	**104.08**	**27.75**	**7.07**	1.35	**19.15**	1.52
C_i	**25.35**	0.64	**11.30**	**39.58**	**3.07**	**5.24**	**3.66**
E	**6.31**	**135.58**	**59.30**	**19.46**	1.16	**17.20**	1.25
F_v/F_m	**29.34**	**49.06**	**5.96**	**19.26**	**5.53**	**3.12**	**3.82**
Y(II)	**38.12**	2.21	**4.05**	**32.68**	**4.93**	**5.16**	2.31
Y(NPQ)	0.66	**12.34**	**22.18**	**6.83**	**3.00**	0.59	0.81
Y(NO)	**14.48**	**18.81**	**8.03**	**15.86**	2.19	2.77	2.67
ETR	**21.59**	**128.81**	**19.25**	**10.70**	0.54	**17.61**	1.66
Chl *a*+*b*	**11.43**	**27.98**	**19.02**	**12.56**	0.66	2.23	1.73
Carotenoids	**31.09**	**45.87**	**20.88**	**23.84**	**4.62**	**10.19**	2.55
Glucose	1.76	**7.28**	**3.15**	1.12	0.36	2.26	0.48
Fructose	2.97	2.08	2.17	1.78	1.36	0.06	0.40
Sucrose	**8.32**	**26.16**	**4.07**	3.91	1.50	1.72	1.63
Starch	**18.03**	2.03	**4.74**	**9.52**	1.75	**5.64**	2.72
MDA	**10.19**	**73.67**	**3.82**	**11.56**	0.75	**14.27**	1.22
H_2O_2	**6.26**	**51.65**	**20.69**	**6.71**	**2.98**	**6.54**	1.35
$O_2^{\bullet -}$	**16.55**	**83.57**	1.06	**15.60**	1.69	**11.39**	1.80
SOD	**25.94**	2.05	**97.98**	**6.12**	1.22	**3.28**	0.95
APX	3.83	**103.23**	**15.93**	0.06	**4.29**	**16.25**	0.67
CAT	**21.09**	**44.83**	**30.02**	**11.79**	2.72	**21.81**	0.29
GR	1.66	0.09	**4.41**	0.03	**3.91**	**38.09**	**3.52**
IGL	**605.22**	**346.10**	**552.93**	**521.24**	**226.65**	**318.52**	**547.09**
CHS02	**455.89**	**416.65**	**243.43**	1.84	**73.54**	**441.98**	**269.13**
PR1	**15.23**	**1640.19**	**485.40**	**4.10**	**9.35**	**281.32**	**12.79**
PAL	**248.36**	**272.56**	**178.72**	**640.09**	**125.68**	**251.93**	**248.84**
LOX3	**703.29**	**1575.64**	**1086.47**	**706.18**	**363.10**	**458.14**	**382.61**
CHI	**880.89**	**7865.54**	**2222.63**	**553.67**	**704.37**	**2026.00**	**757.34**
GLU	**856.29**	**2431.59**	**200.44**	**259.86**	**209.98**	**238.50**	**353.30**
Bm	**1347.40**	-	**521.55**	-	**223.10**	-	-

*Bold values are significant ($P \leq 0.05$)

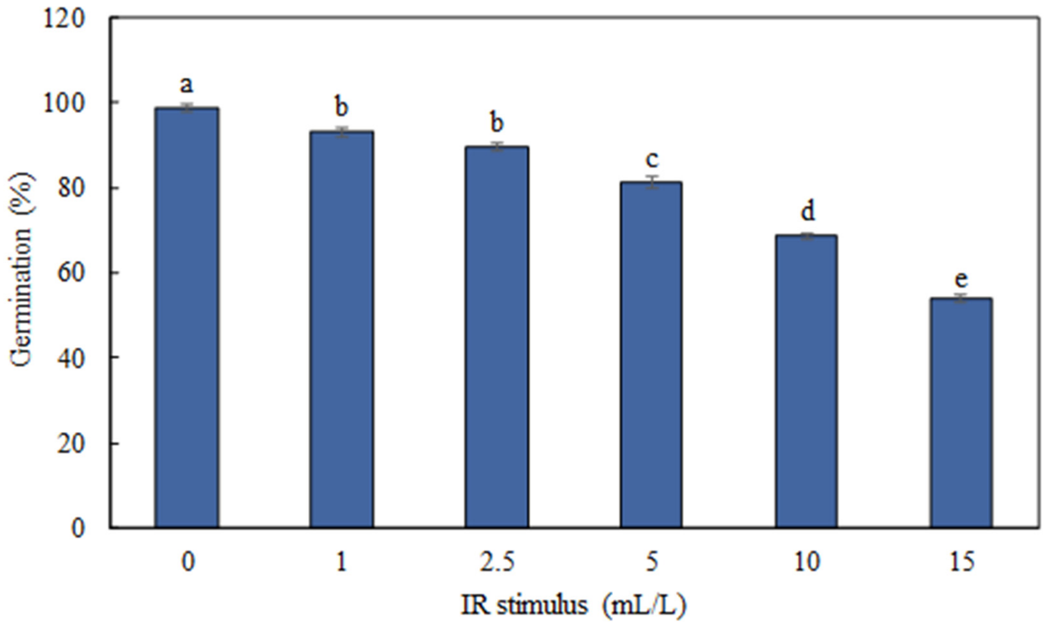
120
100
80
60
40
20
0
Germination (%)
a
b
b
c
d
e
0
1
2.5
5
10
15
IR stimulus (mL/L)

Figure S1
